# Effect of maternal status and breastfeeding practices on infant nutritional status - a cross sectional study in the south-west region of Bangladesh

**DOI:** 10.11604/pamj.2013.16.139.2755

**Published:** 2013-12-12

**Authors:** Mohidul Islam, Shahinur Rahman, Kamruzzaman, Mominul Islam, Abdus Samad

**Affiliations:** 1 Dept of Applied Nutrition and Food Technology, Islamic University, Kushtia-7003, Bangladesh

**Keywords:** Breast feeding, Low birth weight, maternal anthropometry, maternal socio-demography, infant growth

## Abstract

**Introduction:**

The aim of this study was to address the current scenario of LBW and infant nutritional and to analyze the effect of maternal status and pattern of their breast feeding practices on perinatal and postnatal infant development.

**Methods:**

Cross-sectional study design with structured questionnaires was used among 510 mother-infant pair to collect data. Maternal anthropometric, socio-economic and demographic characteristics and breast feeding practice were used as independent variable and birth weight and infant growth status as dependent variable. Descriptive and crosstab analysis were used to analyze the effect.

**Results:**

The study revealed that about 29.4% infants were born with low birth weight (LBW). Mother with no education and from low income family were more likely (OR: 3.484, 95%CI: 1.993-6.089 and OR: 2.078, 95% CI: 1.274-3.387) to have LBW infant compared with mother with higher education and from higher income family. Similarly, lower maternal height, weight and MUAC (< 150 cm, <50 kg and < 22 cm respectively) were shown to have more risk of having LBW compared with higher height, weight and MUAC (RR: 1.628, 2.375 and 2.115; 95%CI: 1.250- 2.120, 1.844- 3.059, 1.623- 2.757). The prevalence of exclusive breastfeeding was found among 45% mother. Postnatal growth and development of infant was not found significantly different (P > 0.05) among those who breast feed exclusively and non-exclusively.

**Conclusion:**

The study confirms that lower level of maternal education; family income and anthropometric measurement significantly increase the risk of LBW. The prevalence of exclusive breastfeeding practice was not found satisfactory.

## Introduction

Low birth weight (LBW) is considered as birth weight less than 2500gm and in Bangladesh, 45% child born with LBW [1, 2]. It has been speculated that low birth weight predisposes children to a high risk of diabetes, heart diseases and other chronic conditions later in life [3–8]. Data cited in National Food and Nutrition policy (1997) document shows that LBW ranges from 30-50 percent, however from different other studies it has been revealed that LBW prevalence rate is about 30 percent [9, 10]. It is assumed that, under nutrition, both before and during pregnancy, causes intrauterine growth retardation and is one of the major reasons for the high LBW. Between 1990 and 2004, underweight levels among children fell from 67% to 48% and child stunting fell from 66% to 43% [11, 12], but the levels are still unacceptably high. Some studies, from Bangladesh, showed that mean birth weight increase with an increase in mother's age from 14 to 31 years, while after 32 years birth weight decrease and the highest mean birth weight occurred in women between 26 and 31 years of age [2]. There are studies both in support and to deny the relationship between maternal socioeconomic and anthropometric characteristics and birth weight [13–15].

Breastfeeding is considered as the pivotal factor between life and death for the vast majority of children in developing countries, but pattern of breast feeding and exclusive breast feeding is more important, which is ignored often by most mothers. Breast milk is a natural resource that has a major impact on infant's health, growth and development and it is recommended for at least the first two years of a child's life [16]. Breast milk contains all desirable nutrients in right quantity that a baby needs and are easily digestible [17]. However it is important to ensure exclusive breastfeeding for the first six months of life, only 35.0% of infants worldwide were exclusively breastfed during the first six months of life and 27.0% under six months infant were bottle fed [16]. It is evident that inappropriate breastfeeding practices are associated with severe malnutrition in the under five children, lack any advantage in terms of weight gain and are associated with growth faltering [18]. A study conducted in Khulna, Bangladesh, stated that the prevalence of breastfeeding as 96% [19], but the prevalence of exclusive breastfeeding and the effect of breastfeeding on infant nutritional status were not clearly stated. Early initiation of breastfeeding generally lengthens the duration of breastfeeding and also prevents postpartum hemorrhage [19], however the pattern of initiation of breastfeeding was not clearly reported in Bangladesh.

In developed countries during the last two decades a large number of studies have been conducted to identify the effect of maternal status and breastfeeding practices on infant development. In Bangladesh, few studies have been conducted to find out the effect of maternal status and breastfeeding practices on infant nutritional status and most of them are weak due to inappropriate study design and lack of statistical validity. We think it is important to investigate the relationship between these in a view to prevent infant morbidity and mortality. The objectives of this analysis was to determine how maternal socioeconomic and anthropometric status effect on birth weight, particularly in determining low birth weight (<2500 gm), and how breastfeeding practice, particularly the type and effective time of initiation and termination of breastfeeding effect on infant growth and development.

## Methods

### Subjects

A total of 510 mother-infant pairs were included and the study was continued for 6 months. Mothers and their infants attending the selected hospitals and maternal clinics in Kushtia during the study period were regarded as the study population. Infants’ of up to 24 months of age was selected and infants above 24 months of age were excluded from the study. The mothers were selected beyond any ages. Some selection and exclusion criteria were followed as women whose health status was normal and who gave normal live birth were included and women with any severe health complication and caesarian cases were excluded from the study.

### Study design

The cross-sectional study was conducted during the period of September 2011 to February 2012. A detailed questionnaire was used to collect data on maternal and infant characteristics. Maternal variable as maternal age, maternal education level and maternal anthropometry like maternal height, weight and mid upper arm circumference (MUAC) was used as maternal variable. Family income was also used as a variable. Whenever mothers come to maternal clinic were interviewed and investigated. Maternal age, level of education and family income was collected from direct questionaries’ and from hospital log book; maternal health card was used to collect maternal anthropometric measurement during pregnancy. Additionally maternal height and MUAC was also measured during the interview period according to standardized techniques [20]. A non-stretchable tape made of fiberglass was used to measure maternal MUAC. All of the maternal variables were categorize into several category (Table 1) to aid in data processing and evaluation of specific effect on infant birth weight. The entire maternal characteristics were used as independent variable. Infant birth weight was used as dependent variable. Birth weight of every infant was collected from maternal and infant health cards and from visited hospital's log book. Birth weight was categorized into three categories (Table 1) as < 2500 gm, 2500-2999 gm and = 3000 gm. According to WHO cutoff points < 2500 gm of birth weight was considered as Low Birth Weight (LBW) [21]. Details data on the pattern of breast feeding practice was also collected during the interview session. The women were asked about their current and previous infant feeding practices, including the time of initiation and duration of breastfeeding, use of formula, bottle feeding and introduction of complementary food. Use of any pre-lacteal feeding was also documented and categorize into another group. Exclusive breast feeding (EBF) up to six months was categorized into one category and EBF for less than six months was categorizing into other category. The other categories of breast feeding practice were as breast feeding on demand and continuing breast feeding for 24 months. Three infant variables were used as Stunting, Wasting and Stunting and Wasting. Infant anthropometric measurements were done during the period of interview. Infant age was collected from infant health card or from questionnaire. A research team consisting of students and teacher were directly involved in data collection in the maternal clinic. Data were collected with the help of health assistant working in the maternal clinic, Infant weight and height was measured using electronic scale and wooden measuring board following standard measuring procedure [20]. Before measurement, the scales were calibrated and validated by the research team. Waterlow classification was used to classify infant into normal, wasting, stunting and wasting and stunting. In Waterlow classification% height for age and% weight for height was calculated using standard reference data for height of normal infant of the same age and weight of normal infant of the same height [20].

**Table 1:** Relative distribution of infant according to their birth weight and categories of maternal variables.

Variable	Total	Birth Weight (gm)		
		<2500 (LBW*)	2500-2999	≥3000
	*n (%)*	*n (%)*	*n (%)*	*n (%)*
**Maternal Age (years)**				
Up to 20	110(21.57)	34(30.9)	48(43.6)	28(25.5)
21-25	282(55.29)	80(28.4)	112(39.7)	90(31.9)
26-30	55(10.78)	13(23.6)	20(36.4)	22(40)
≥ 31	63(12.35)	23(36.5)	23(36.5)	17(27)
**Maternal Education**				
Illiterate	58(11.37)	32(55.2)	15(25.9)	11(18.9)
Primary	75(14.71)	35(46.7)	32(42.6)	08(10.7)
class 6- S.S.C*	272(53.33)	69(25.3)	11542.3)	88(32.4)
H.S.C*	52(10.20)	11(21.2)	26(50)	15(28.8)
Graduation & above	53(10.39)	03(5.7)	1528.3)	35(66)
**Family Income (Tk)**				
Up to 3999	81(15.88)	35(43.2)	28(34.6)	18(22.2)
4000-7999	135(26.47)	42(31.1)	62(45.9)	31(23.0)
8000-11999	119(23.33)	36(30.1)	46(38.7)	37(31.1)
12000-15999	87(17.05/	25(28.7)	32(36.8)	30(34.5)
≥16000	88(17.25)	12(13.6)	3539.8)	41(46.6)
**Maternal Height (cm)**				
Up to 144.9	74(14.51)	32(43.2)	22(29.8)	20(27)
145-149.9	89(17.45)	33(37.1)	28(31.5)	28(31.4)
150-154.9	101(19.8)	27(26.7)	46(45.5)	28(27.8)
155-159.9	118(23.14)	32(27.1)	52(45.7)	32(27.7)
160-164.9	61(11.96)	13(21.3)	25(41)	25(37.7)
≥165	67(13.14)	13(19.4)	28(41.8)	26(38.8)
**Maternal Weight (kg)**				
Up to 45.9	57(11.18)	30(52.6)	10(17.5)	17(29.8)
46-49.9	75(14.71)	38(50.7)	22(29.3)	15(20)
50-54.9	120(23.53)	30(25)	53(44.2)	37(30.8)
55-59.9	106(20.78)	30(28.3)	49(46.2)	27(25.5)
≥60	152(29.8)	22(14.5)	69(45.4)	61(40.1)
**Maternal MUAC (cm)**				
≤ 22.0	75(14.71)	41(54.7)	25(33.3)	09(12.0)
22.1-24	118(23.14)	42(35.6)	51(43.2)	25(21.2)
24.1-26	133(26.08)	33(24.8)	55(41.4)	45(33.8)
26.1-28	83(16.27)	19(22.9)	31(38.6)	33(39.7)
28.1-29	50(9.8)	10(20.0)	18(36.0)	22(44.0)
>29	51(10)	05(9.8)	23(45.0)	23(45.1)
**Total** ***(n%)***	**510(100)**	**150(29.4)**	**203(39.8)**	**157(30.8)**

* LBW = Low Birth Weight, S.S.C. = Secondary School Certificate, H.S.C. = Higher Secondary School Certificate.

### Statistical analysis

Maternal variables were also categorized into different groups. Descriptive statistics were used to analyze the relative distribution of birth weight in different categories of maternal variable. Each of the maternal independent variable was dichotomized into two groups as up to 20 years and more than 20 years for age; uneducated and educated and so on (Table 2) and coded as 0 and 1. Maternal MUAC was dichotomized into = 22 cm and > 22 cm and maternal MUAC = 22 cm was considered as poor maternal nutrition [22]. Dependent variable infant birth weight was also dichotomized into two groups as low birth weight and normal birth weight and coded as o and 1 respectively. Chi square test was used to assess the relationship between dependent and independent variable. Odd Ratio and Relative Risk with 95% confident interval (CI) were calculated using crosstab, to measure the magnitude of odd and risk of LBW and infant growth retardation in terms of wasting and stunting. To interpret the relationship between pattern of exclusive breastfeeding and infant nutritional status, two (02) important maternal age groups, 26-30 years and (=31 years were selected. Infant nutritional status was also dichotomized into two groups as wasting and normal; stunting and normal and wasting and stunting and normal and codded as o and 1 respectively. Breastfeeding pattern was also dichotomized into two groups as exclusive breast feeding and non-exclusive breast feeding. Association between pattern of breast feeding (independent variable) and infant nutritional status (dependent variable) was also assessed by calculating Chi square and OR with 95% CI using crosstab. All analysis was done using SPSS software, version 15.0 (SPSS Inc, Chicago, IL, USA). P value of <0.05 was considered to be significant.

**Table 2:** Independent effects of demographic, socio-economic and anthropometric risk factors for risk for low birth weight (LBW) analyzed by binary logistic regression.

Socio-demographic Factors	Pearson Chi-Square	*P* Value	OR* (CI 95%)
**Maternal Age:** (≤20 years vs. >20)†	0.151	0.697	1.095 (0.693- 1.732)
**Maternal Education:** (Illiterate vs. Literate)†	20.917	0.000	3.484 (1.993-6.089)
**Family Income (Tk):** (≤ 3999 vs. ≥4000)†	8.830	0.003	2.078 (1.274-3.387)
**Maternal Height:** (<150 cm vs. ≥ 150)†	12.639	0.000	2.044 (1.374- 3.043)
**Maternal Weight:** (<50 kg vs. ≥ 50 kg)†	41.910	0.000	3.835 (2.520- 5.837)
**Maternal MUAC:** (≤ 22 cm vs. ≥ 22cm)†	24.817	0.000	3.385 (2.058- 5.567)

* OR: Odd Ratio.

† Maternal age > 20 years, Literate, Family income ≥ 4000 Tk, Maternal height ≥ 150 cm, Maternal weight ≥ 50 kg and maternal MUAC ≥ 22 cm was considered as reference categories.

### Ethical approval

This research was approved by the ethics committee of the faculty of Applied Science and Technology of Islamic University, Kushtia, Bangladesh. The objective of this study and study protocol were first described to the subjects and oral consent to participate in the study was taken. All of the maternal clinics that took part in this study also gave their approval.

## Results

### Maternal characteristics and infant birth weight

**Figure 1:**
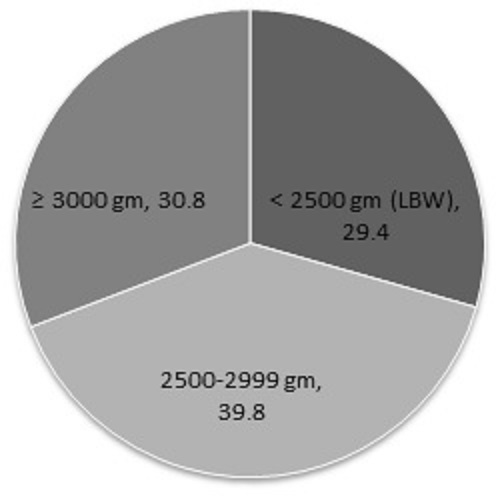
Distribution of infants according to birth weight

**Figure 2:**
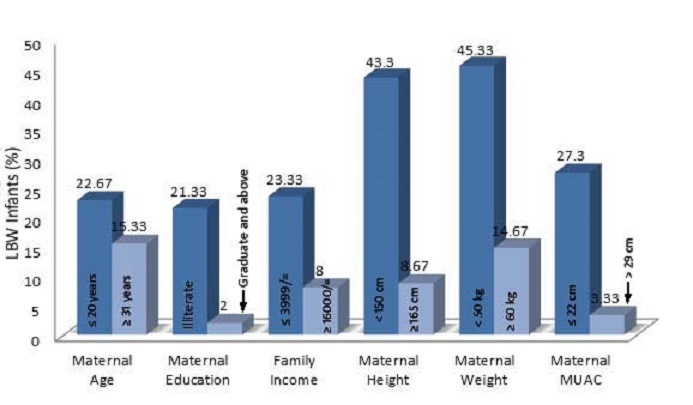
Infants with LBW according to category of risk factors (Total number of LBW infants = 150)

According to maternal age category, highest percentage (36.5%) of low birth weight infant was found in the age group of = 31 years; however the lowest percentage (23.6%) was found in the 26-30 age group (Table 1, Figure 1, Figure 2). 40% infant were in the age group of 26-30 year with birth weight greater than 3 kg, which was the highest among all of the maternal age and birth weight category. However only 11.37% mother-infant pair were in the illiterate mother group, highest percentage (55.2%) of LBW infant were in this group, following 46.7% in the group where mother has completed primary education. In contrast, mothers, who were graduated, were shown to have the highest percentage (66) of infants with normal weight and only 5.7% of infant with LBW. According to family income category the lowest family income category (≤ 3999) were with highest percentage (43.2%) of low birth weight infant, whereas the lowest percentage (13.6%) of the LBW were in the highest family income category (≥ 16000). Percentages (46.6%) of normal weight infant were also high in the highest family income group. According to maternal height and weight category the highest percentage of LBW infant (43.2 and 52.6% respectively) were found in the lowest maternal height (<145 cm) and weight (< 46 kg) category respectively. Mother with mid upper arm circumference < 22 cm were tend to give highest percentage (54.7%) of LBW infant. As a total, 29.4% of infant were with LBW.

Except age (p = 0.697), all of the maternal independent variable were significantly (p ≤ 0.003) associated with LBW (Table 2). Mother with age ≤ 20 years were associated with similar Odd of having LBW (OR: 1.095, CI: 0.693- 1.732) as compared with mother with age greater than 20 years. Illiterate mother were shown to have more than 3 times more odd (OR: 3.484, CI: 1.993-6.089; P = 0.000) of having low birth weight infant than those of educate mother. Mothers from low income family (< 3999/= BDT) were also tend to have more (OR: 2.078, CI: 1.274-3.387; p = 0.003) LBW babies than mothers from higher income family (>16000/= BDT). Similarly maternal height, weight and MUAC also showed a significant effect (p = 0.000) on LBW. Mother weighted less than 50 kg were associated with 383% (CI: 2.520- 5.837) more odd of having LBW than mother with higher weight. Mother with MUAC < 22 cm also shown similar result (OR: 3.385, CI: 2.058- 5.567; p = 0.000).

### Breast feeding practice and infant nutritional status

Among the studied mother-infant pair 61.1% mother were shown to initiate breastfeeding within 1 hour of child birth, while the rest of the mother (38.1%) did not do that (Table 3). The study showed that 45% mother breastfeed their child exclusively for the first six month, whereas 90% mother gives breast milk to their child on demand. 97.8% of studied mothers were shown to have a wish to continue the breastfeeding up to 2 years of infant's age. Only 21% of studied mothers were shown to give pre-lacteal feed. Honey was the most common among the pre-lacteal feeds. Analysis of the association of exclusive breast feeding practice with infant nutritional status were shown that among 25 exclusively breastfed infant only 2 were found with wasting; while in the case of 33 non-exclusively breastfed infant the number was 6 (Table 4). Using Waterlow classification, weight and height of each infant was compared with the reference infant's weight and height with same age. The highest percentages (26.7%) of nutrition related complication was growth retardation which was among non-exclusively breastfed infant and the second highest was both wasting (20%). Among exclusively breast fed infant only 16% were stunted, whereas only 8% and 4% were wasted and both stunted and wasted respectively. In the case of greater than 30 years of maternal age group, 52.6% infants were stunted among nonexclusively breastfed which was the highest percentage both in exclusively and non-exclusively breastfed infant. The percentages of all form of malnutrition related complication were higher among non-exclusively breast fed infant than exclusively breastfed infant. The crosstab analysis were shown that all of the association in both maternal age groups was non-significant (p > 0.05), except only the association of wasting with breastfeeding practice (p< 0.05). However the odd and risk (Table 5) of all types of malnutrition related complication were higher among non-exclusively breastfed infant, but was not found statistically significant (1 was included in the 95% CI).

**Table 3:** Pattern of Breastfeeding practice

Breast Feeding Practice	Total Number (%)	Positive *n (%)*	Negative *n (%)*
Initiated breastfeeding within one hour of child birth.	510 (100)	312(61.1)	198(38.9)
Give pre-lacteal feeds.	510 (100)	107(21)	403(79)
Breastfed on child demand.	510 (100)	464(90.9)	46(9.1)
Children that Breastfed Exclusively (EBF) for the first six months of life.	510 (100)	230(45)	280(55)
Continuing and will continue breastfeeding for 2 years.	510 (100)	499(97.8)	11(2.2)

**Table 4:** Prevalence of wasting Stunting and wasting and wasting according to pattern of exclusive breastfeeding practice

Breast Feeding Status		Maternal age Group 26-30 years					
		Wasting		Stunting and wasting		Stunting	
	Number *(%)*	Present *n(%)*	Absent *n(%)*	Present *n(%)*	Absent *n(%)*	Present *n(%)*	Absent *n(%)*
Exclusive Breast Feed Infant	25 (100)	02 (8)	23 (92)	01 (4)	24 (96)	04 (16)	21 (84)
Non-Exclusive Breastfeed Infant	30 (100)	06 (20)	24 (80)	03 (10)	27 (90)	08 (26.7)	22 (73.3)
Total	55 (100)	8 (14.5)	47 (85.5)	04 (7.3)	51 (92.7)	12 (21.8)	43 (78.20)
**Maternal Age Group > 30**							
Exclusive Breast Feed Infant	25 (100)	04 (24)	21 (76)	02 (8)	23 (92)	11 (44)	14 (56)
Non-Exclusive Breastfeed Infant	38 (100)	15 (39.5)	23 (60.5)	07 (18.4)	31 (81.6)	20 (52.6)	18 (47.4)
Total	63 (100)	19 (31.1)	44 (69.9)	09 (14.3)	54 (85.7)	31 (49.2)	32 (50.8)

**Table 5:** Independent Effects of exclusive breast feeding practice for risk for infant growth retardation analyzed by binary logistic regression

Effect	Pearson Chi-Square	*P* Value	OR* (CI 95%)
**Maternal Age Group ≤ 30 years†**			
Wasting (Non-EBF vs. EBF)	1.580	0.209	2.875 (0.526- 15.729)
Stunting and Wasting (Non-EBF vs. EBF)	0.728	0.617	2.667 (0.260- 27.381)
Stunting (Non-EBF vs. EBF)	0.910	0.340	1.909 (0.499- 7.298)
**Maternal Age Group >30 years†**			
Wasting (Non-EBF vs. EBF)	3.945	0.047	3.424 (0.979- 11.969)
Stunting and Wasting (Non-EBF vs. EBF)	1.337	0.298	2.597 (0.493- 13.677)
Stunting (Non-EBF vs. EBF)	0.450	0.503	1.414 (0.513- 3.900)

* OR: Odd Ratio.

† Exclusive Breast Feeding (EBF) was considered as reference category

## Discussion

The study was conducted on sample of 510 mothers and their infants selecting randomly to assess the effects of maternal status and breastfeeding practices on infant development. Then we selected some factors randomly to identify the effect of maternal status on infant development. Two types of characteristics of mothers were studied against different ranges of birth weight of infant to identify the effect of maternal status on infant and the effect of the pattern of the exclusive breast feeding practices on infant nutritional status were studied.

In Asia the prevalence of LBW is highest and in South-East Asia one third of the newborn are with LBW [23]. The current study showed the prevalence of LBW as 29.4%, however according to study of Bangladesh Bureau of Statistics the prevalence was 36% in Bangladesh [24]. A study reported the rate of LBW as 31.2%, studied on 1000 mothers in Dhaka Medical College [21]. A study conducted in Bangladesh reported that, 15.9% mothers were found to be under 20 years and 8.9% were above 30 years of age [10]. The current study also revealed that the percentage of mother-infant pair were highest in the 21-25 age groups. About 11.37% of mothers had no formal educational background, 14.71% had primary level, 53.33% had six to S.S.C level, 10.2% had higher secondary and 10.39% completed graduation. There is some dissimilarity in educational status in comparison to the study conducted by Karim and Taylor (1997) [10].

It was revealed from the logistic regression analysis that maternal age is not an important predictor of LBW. However the percentages of LBW infant in the maternal age group of up to 20 years and more than 30 years were little bit higher than the age groups of 21-30 years though was not significant. Thus maternal age ranging from 21-30 years was found to be most suitable age group for giving birth with normal birth weight. The findings of the present study were in agreement with many similar studies both in developed and developing countries [2, 9, 10, 25]. When maternal age groups were dichotomized into to two groups for binary logistic regression analysis for the effect of maternal age < 20 years on LBW did not show any significant effect (p = 0.697). Mother's educational status has great influence on birth weight of the infant. The present study showed a decreasing trend of the percentage (55.2% - 03%) of LBW with increasing maternal educational status. Maternal education was shown to be an important predictor of LBW. Literate mother were shown to give less number of babies with LBW. Many other studies have shown similar findings [9, 10, 26]. It is possible that the effect of education on birth weight is to some extent a reflection of other influences like maternal nutrition, which can also be enhanced by better income opportunities for educated couples. The highest percent (26.47%) of mother-infant pair were in the family income group of 4000-7999 BDT. Family income was associated with LBW in a similar trend of decreasing percentage of LBW with increasing family income. In the present study, it was revealed that the highest incidence of LBW (43.2%) was found among the families having income less than 4000 BDT and the lowest LBW (13.6%) among the families with income equal to or greater than TK. 16000 BDT. Family income less than 4000 BDT showed a higher Odd (Odd: 2.078, CI: 1.274-3.387) of having LBW than more family income and the association was significant (p = 0.003). Others studies reported similar findings in Bangladesh [21, 27] and other countries [28, 29]. Mother in a family with higher income usually gets proper nutrition and better care than a mother in low income family.

The magnitude of effect of maternal weight and maternal MUAC were more intense than the maternal height but statistically significant. Regression analysis showed that women with height and weight < 150 cm and < 50 kg respectively were more likely to have 2 and 3 times more LBW respectively than women with more height and weight. A similar study using cut-off points for low maternal weight at < 45 kg was shown similar findings of having (OR: 3.51, 95%CI: 1.74-7.15) LBW; however the same study shown a non-significant association between maternal height and LBW using a cut-off point of < 145cm [22]. Some other study reported significant association between maternal height and LBW [30, 31], while some others study report were non-significant [32]. However the other study findings were controversial, the current study confirms maternal weight and height as strong predictor of LBW. Mother with MUAC < 22 cm were more than 3 times likely to have LBW babies as compared to mother with MUAC SUPER_OR_EQUAL 22cm and the association between these variables was found statistically significant (p = 0.000). Ojha and Malla in 2007, Husaini et al. in 1995 and Neumann et al. in 1995 also reported positive association between maternal MUAC and LBW, though different cutoff-points were used [22, 33, 34]. MUAC is usually reflected by the nutritional status. Mother with MUAC > 22 cm were tends to give more babies with normal birth weight. Mother with MUAC < 22 cm was negatively associated with birth weight of infants.

Since the time immemorial mammalians babies were feeding with their mother's breast milk. Breast milk is considered to be the only and ideal food for the infants for the first six months of life and exclusive breast feeding for the first six months of life is an important strategy for the proper growth and development of infants. In spite of an increased focus on the necessity to ensure exclusive breast feeding for the first six months of life, the percentage of exclusive breast feeding is not appreciable both in developed and developing country. However the practice of feeding colostrum has improved in the past decade (87%) and 43% of children were exclusively breastfeed for the first six months of life [35], whereas 45% of mother was found to breast feed exclusively in our current study. More than 97% of studied mother continued and wish to continued breastfeeding for up to 2 years, which is certainly a positive sign for breast feeding practice. The use of honey and other sweet item as pre-lacteal feeding were more common as a cultural believe that the first milk is dirty and as a believer to give honey. The potential contamination for pre-lacteal feeding are more and there are chance of developing allergic manifestation in the future life [36, 37]. However several studies showed higher percentage (79-93%) of giving pre-lacteal feeding [38, 39], current study showed to have only 21% to do this practice. According to WHO, it is essential to initiate breastfeeding within half an hour after birth [40]. Early initiation of breastfeeding helps to ensure proper nutrition and first defense for the infant and help to secrete milk [41] and initiate a positive bonding between mother and infant [42]. However only 22% of mothers were found to initiate breastfeeding within 1 hour as reported by Sayed Mahmood et al., 61.1% mother were found to do this in the current study, [43]. To identify the effects of exclusive breastfeeding practices on infant development, two important maternal age groups were selected. However beneficial effect of exclusive breastfeeding has been supported by several studies [44–46], the current study did not show any effect of exclusive breastfeeding on child growth, compared with non-exclusive breastfeeding using crosstab analysis. The odd of having under nutrition in non-exclusively breast feed infant was higher than in exclusively-breast feed infant, however the association between breast feeding practice and nutritional status of infant was found non-significant. This might be due to small sample size (55 and 63).

## Conclusion

Low birth weight is a common problem in developing world where malnutrition effect particularly on maternal nutrition. This study identified that lower level of maternal education and anthropometry as well as family income increases the risk of LBW. This indicates increased level of maternal education ensures maternal awareness and higher income help to ensure proper food, which is essential for proper maternal nutrition and perinatal infant development. Practices of exclusive breastfeeding were found less than half of the studied mother. It is important to ensure exclusive breastfeeding practice for all of the mothers. Though in the current study, Exclusive breastfeeding up to 6 months of infant's age were shown to have no effects on infant's nutritional status, exclusive breast feeding is important for infant nutrition. Practice of EBF was not found satisfactory in the studied mother infant pairs. Measure should need to ensure exclusive breastfeeding up to six months and early initiation of breastfeeding within one hour.
